# Towards a good environmental status: a 4-year monitoring study on the contamination of the Bay of Luebeck with energetic compounds prior to munitions remediation

**DOI:** 10.1007/s00204-025-04027-x

**Published:** 2025-04-24

**Authors:** Tobias H. Bünning, Jennifer S. Strehse, Edmund Maser

**Affiliations:** Institute of Toxicology and Pharmacology for Natural Scientists, University Medical School Schleswig-Holstein, Brunswiker Str. 10, 24105 Kiel, Germany

**Keywords:** Energetic compounds, Trinitrotoluene, Explosives, Blue mussels, Dumped munitions, Nitroaromatics, Monitoring study, Baltic Sea

## Abstract

In the Bay of Luebeck, two out of several munition dumping areas in the German Baltic Sea are located, where approximately 65,000 t of munitions were dumped in the post-World War II period. The explosives used in these munitions, such as the nitroaromatic compound 2,4,6-trinitrotoluene (TNT) and its metabolic transformation products 4-amino-2,6-dinitrotoluene (4-ADNT) and 2-amino-4,6-dinitrotoluene, (2-ADNT) are considered mutagenic and carcinogenic and pose a potential threat to marine ecology and human health when they leak from corroding shells into the surrounding water. A 4-year pilot monitoring program, conducted in collaboration with the Ministry of the Environment of Schleswig–Holstein, aimed to assess the current contamination level of the Bay of Luebeck’s waters with various energetic compounds (EC) from dumped munitions and to evaluate the feasibility of integrating these investigations into the monthly routine sampling program of Schleswig–Holstein's coastal waters. This routine water sampling was expanded by direct monitoring of specific munition dumping sites in the Bay of Luebeck. Beyond repeated water samples, these specific dumping areas were long term monitored by using blue mussels and passive sampler systems which both are ideal approaches to infer whether these compounds are entering marine ecosystems such as in the Bay of Luebeck. In all water samples from the routine program collected monthly at four locations from the seabed and surface, TNT and six other EC were detected. However, only 1,3-dinitrobenzene (1,3-DNB), 2,4-dinitrotoluene (2,4-DNT), and 1,3,5-trinitro-1,3,5-triazine (RDX) were measured at average concentrations exceeding 1 ng/L. As expected, TNT water concentrations at the specific dumping arears were slightly higher (by a factor of 2–4) compared to the routine monitoring sites. At the same locations, EC were detected in a few individual blue mussel samples, with all concentrations remaining below 0.6 ng/g dry weight. EC concentrations in the passive samplers were in the one or two-digit nanogram range per passive sampler, except for 1,3-DNB which reached up to 105 ng per passive sampler. As a conclusion, over the course of the last 3 years, it became apparent that EC are ubiquitous distributed in the Bay of Luebeck, but their concentrations are still relatively low, even in both specific dumping areas.

## Introduction

**Fig. 1:**
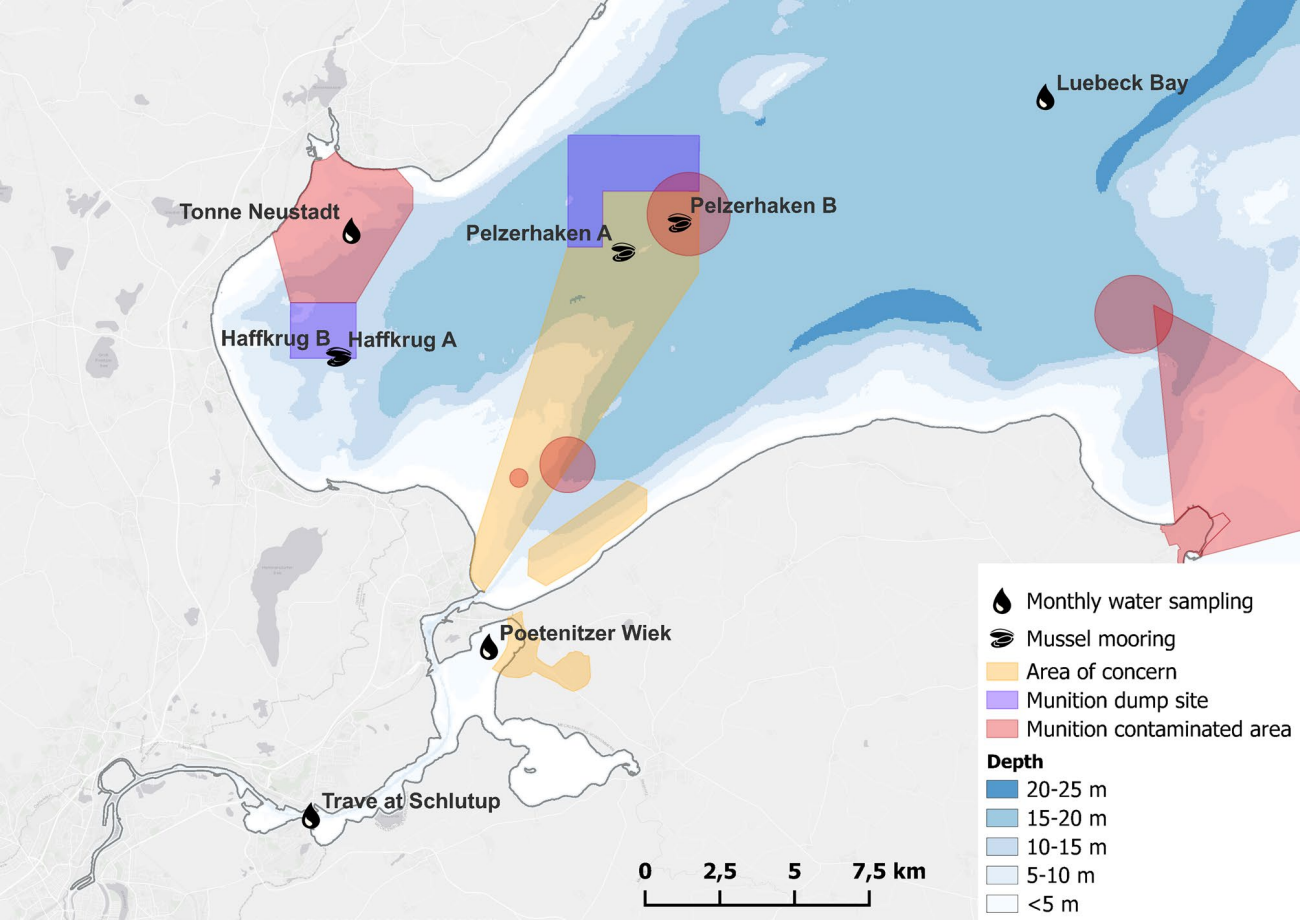
Map of the Bay of Luebeck with the munition dump sites Haff-krug and Pelzerhaken as well as the areas contaminated by munitions and the suspected munitions sites (areas of concern). The positions of the monthly water sampling and the positions of the blue mussel moorings are shown

After the end of World War II, vast amounts of unused munitions remained in Germany. Rapid demilitarization and disarmament of Germany were among the top priorities of the Allied occupiers, and marine dumping was seen as the simplest way to achieve this goal. As early as in June 1945, areas in the German North and Baltic Seas were designated for the disposal of all types of conventional munitions. In the Bay of Luebeck, two out of several areas in the Baltic sea, were designated by the British military government: Haffkrug to the west, approximately 2.16 km from the shore, and Pelzerhaken more eastern, 2.1 km to shore (Fig. 1, labeled as “Dumping Area”). Over 60,000 t of conventional munitions of all calibers were dumped there between June 1945 and January 1949 (Böttcher et al. 2011)—approx. 50,000 t at Pelzerhaken and 10,000 t at Haffkrug with an overall uncertainty of 5000 t (LASH 1960). However, since the dumping did not take place exclusively in the two designated areas but was partially carried out along the way (“on-route dumping”), the routes from the ports of Neustadt in Holstein and Travemuende to these sites are commonly classified as “munition contaminated areas” or considered “suspected munitions areas” (AmuCad.org 2025; Böttcher et al. 2011; Greinert et al. 2024). In addition, there are legacy contaminants from wartime activities. For example, the eastern part of the Poetenitzer Wiek, which served as a seaplane landing area for the Luftfahrzeugamt See in the 1930s and 1940s, is also considered a “suspected munitions area”. Between the years 1955 and 1957, an estimated 16,000 t of munitions were recovered from the Bay of Luebeck by the company Kaus & Steinhausen, leaving an estimated remaining quantity of approximately 50,000 t of munitions (LASH 1960).

**Fig. 2:**
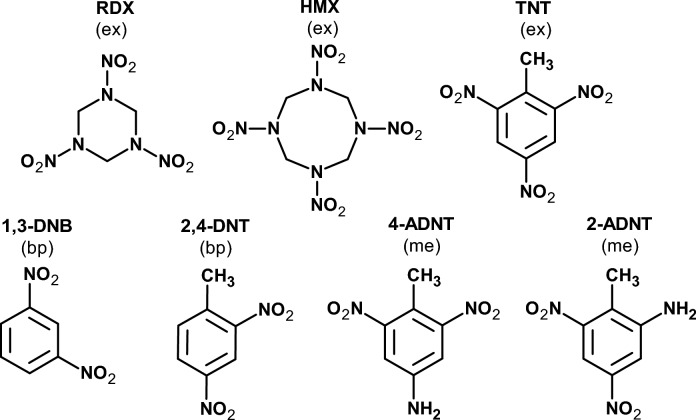
Chemical structures of the explosives (ex) 1,3,5-trinitro-1,3,5-triazinane (RDX); 1,3,5,7-tetranitro-1,3,5,7-tetrazocane (HMX) and 2,4,6-trinitrotoluene (TNT); the explosive production byproducts (bp) 1,3-dinitrobenzene (1,3-DNB) and 2,4-dinitrotoluene (2,4-DNT), and the TNT metabolites (me) 4-amino-2,6-dinitrotoluene (4-ADNT) and 2-amino-4,6-dinitrotoluene (2-ADNT)

The toxic effects of energetic compounds such as 2,4,6-trinitrotoluene (TNT) (Fig. 2) on marine organisms is in the focus of intensive research since several years (Ek et al. 2008; Goodfellow et al. 1983; Lotufo 2013; Lotufo et al. 2010, 2013, 2016; Nipper and Lotufo 2009; Talmage et al. 1999). Moreover, the threads through corroding munitions in the dumping zones of the German Baltic and North Seas have been investigated since 2016 by various national (Environmental monitoring for the delaboration of munitions on the seabed (UDEMM); Trends zur Verbreitung von Munitionsresten in der Meeresumwelt (TATTOO); CONcept for conventional MArine munition Remediation in the German North and Baltic Sea (CONMAR)), and international projects (Decision Aid for Marine Munitions (DAIMON); North Sea Wrecks (NSW); Remediation, Management, Monitoring and Cooperation addressing North Sea UXO (REMARCO)). So far, primary attention has been directed at the Kolberger Heide, located off the mouth of the Kiel Fjord (Appel et al. 2018; Beck et al. 2019; Gledhill et al. 2019; Maser and Strehse 2020; Strehse et al. 2017). This area contains not only large-scale munitions such as moored and bottom mines but also significant quantities of hexanite (“German Schiesswolle”), an explosive that includes TNT as well as other energetic compounds (Fig. 2) (Appel et al. 2018; Strehse et al. 2017). Toxic effects have been observed, among other species, in mussels (Rosen and Lotufo 2007; Schuster et al. 2021; Strehse et al. 2020) and fish such as the common dab (Kammann et al. 2024; Koske et al. 2020; Maser et al. 2024) and pouting (Maser et al. 2023b), which were either caught in the immediate vicinity of munition objects or deliberately exposed to them. In top predators such as eider ducks (Schick et al. 2022), harbor porpoises, and seals, no energetic compounds have been detected so far (Strehse et al. 2024).

Recent surveys in the CONMAR-Project by Greinert et al. using multibeam echo sounder, side-scan sonar and remotely operated and autonomous underwater vehicles equipped for magnetic and photogrammetric mapping have shown that the distribution of munition objects in the Bay of Luebeck extends far beyond the known boundaries of the Haffkrug and Pelzerhaken munition dumping areas (Greinert et al. 2024). Munition objects of all sizes—from small arms cartridges to torpedo warheads and flying bombs such as the Fieseler Fi 103 (“V-1”) were found. The occurrence of EC in the water column of Luebeck Bay was also proven (UDEMM et al. 2019).

Due to the significance of the Bay of Luebeck as a tourist hub and the limited data available on the contamination of its marine environment, this study was conducted in collaboration with the Ministry of the Environment of Schleswig–Holstein (Ministry for Energy Transition, Climate Protection, Environment, and Nature of the State of Schleswig–Holstein) and the State Office for Agriculture, Environment, and Rural Areas (LLUR), which operates under its authority, between August 2019 and August 2022. In addition to monthly routine water sampling in certain areas to determine the seasonally varying release of energetic compounds, passive sampling systems and active biomonitoring with blue mussels was carried out at specific munition dumping sites to investigate the entry of these substances into the marine ecosystem of the Bay of Luebeck.

## Material and methods

### Routine water-sampling program

Monthly water samples were taken from December 2019 as part of the routine water monitoring program by the LLUR at the sampling positions Luebeck Bay and Tonne Neustadt in the Bay of Luebeck, as well as at Poetenitzer Wiek and Trave at Schlutrup in the River Trave which flows into the Bay of Luebeck (Fig. 1, Table 1). Sampling was carried out using a multiple water sampler with five 3.5-L Niskin-bottles and a CTD-unit at 1 m below the surface and 1 m above the ground. Samples were immediately transferred to 1-L ethylene–vinyl acetate (EVA) infusion bags, spiked with 0.25 mL of a 250 ng/mL 1,3-DNB standard solution (until July 2020) or ^13^C^15^N-TNT (from August 2020 onwards) standard solution (in acetonitrile (ACN)) as internal standard and introduced through unconditioned Chromabond Easy solid phase extraction (SPE) columns at ambient temperature in the ship’s laboratory. Due to the use of 1,3-DNB as internal standard until July 2020, 1,3-DNB itself could only be analyzed in samples taken afterwards. Loaded columns were immediately frozen at − 18 °C. The samples were delivered in frozen state to the Institute of Toxicology at Kiel University Medical School (Germany) for chemical analysis by GC–MS/MS and LC–MS/MS. Between April and June of 2020 no sampling could be carried out, due to COVID-19 restrictions.

**Table 1:** Monthly water-sampling positions

Name	Lat	Long	Depth
Tonne Neustadt	N 54° 04.2	E 10° 49.3	8.6 m
Luebeck Bay	N 54° 06.6	E 11° 10.5	21.6 m
Trave at Schlutrup	N 53° 53.6	E 10° 48.0	8.5
Poetenitzer Wiek	N 53° 56.7	E 10° 53.5	4.9

### Blue mussel, passive sampler and specific water monitoring at munition dump sites

**Fig. 3:**
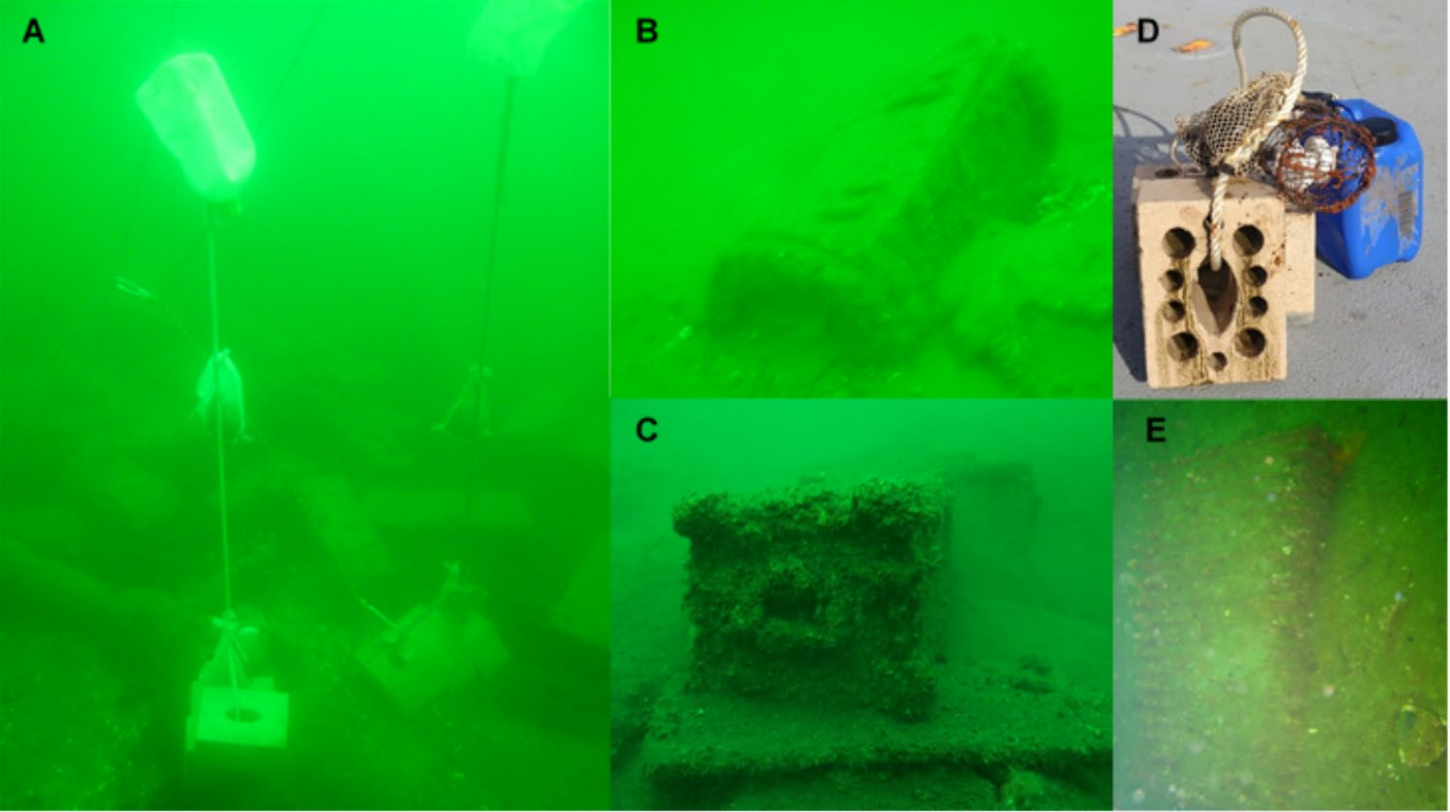
**A** Exchange of the Haffkrug A-mooring in April 2021. Munition crates and objects can be seen in the background; **B** Munition crates at position Pelzerhaken A; **C** Munition crate with unknown contents at position Haffkrug B; **D** Mooring directly after recovery; **E** Unidentified metal object at position Pelzerhaken B. Whether it is munition could not be determined. Photos taken by the scientific diving team of Kiel University

Blue mussels (*Mytilus* spp.) were obtained from the Kiel Marine Farm (Kiel, Germany). Transport of the mussels was carried out in a light-protected and cooled manner, under moist cloths. The maximum time outside the water was approximately 4 h. The deployment of the mussels was carried out by the research diving team of the University of Kiel. For deployment, moorings consisting of an 20 kg anchor made of limestone or concrete, a 1.5-m-long rope, and a 5-L canister as a buoyancy device were used (Fig. 3A and D; Bünning et al. 2021; Strehse et al. 2017). Attached to these moorings, nets with 20 mussels each were mounted directly at the seafloor and 1 m above the seafloor, respectively. Two Chemcatcher passive samplers with HLB membrane and polyether sulfone (PES) protective membrane, which were activated according to the manufacturer’s specifications, were included into the nets directly on the seafloor. In total, mussels and passive samplers were deployed and retrieved five times. Further planned mussel exchanges had to be canceled due to Covid-19 restrictions. Moorings were deployed at two munitions sites each in the Haffkrug and Pelzerhaken munition dumping areas (Fig. 3). The four locations and five exposure periods are listed in Table 2. After recovery, the mussels were frozen directly on board on dry ice and then stored at − 20 °C until they were analyzed. The passive sampler membranes were stored at − 20 °C. The samples were analyzed using GC–MS/MS and LC–MS/MS technology. Beyond the routine water sampling program (see “Routine water-sampling program”), water samples were taken from the surface and near the bottom from the ship at the approximate mooring locations, as well as directly at the mooring positions by divers. Due to the proximity of the two munition piles in Haffkrug (Fig. 3), sampling from the ship was performed only once at the surface and near the bottom, respectively.

**Table 2:** Positions of the mussel moorings, depth, exposure periods, and type of area

Station	Lat	Lon	Depth	Deployed/recovered	Type of area
Pelzerhaken A	N 54° 04.3	E 10° 59.3	20.3 m	19.11.19–11.08.20 11.08.20–17.11.20 17.11.20–03.03.21 03.03.21–27.04.21 27.04.21–14.06.21	Munition contaminated area
Pelzerhaken B	N 54° 03.8	E 10° 57.6	23.8 m	17.11.20–27.04.21 27.04.21–14.06.21	Suspected munitions area
Haffkrug A	N 54° 01.9	E 10° 48.8	17.6 m	18.11.20–04.03.21 04.03.21–28.03.21 28.04.21–15.06.21	Munition dumping area
Haffkrug B	N 54° 01.9	E 10° 48.9	17.6 m	18.11.20–04.03.21 04.03.21–28.04.21 28.04.21–15.06.21	Munition dumping area

### Chemical analysis

#### Materials and chemicals

The following EC standards for calibration were obtained from AccuStandard, New Haven, USA: 1,3-dinitrobenzene (97.0% purity, 1 mg/mL, in acetonitrile (ACN):methanol (MeOH) 50:50), 1,3,5-trinitro-1,3,5-triazinan (99.2% purity, 1 mg/mL, in ACN:MeOH (Methanol) 50:50), 1,3,5,7-tetranitro-1,3,5,7-tetrazocane (99.0% purity, 1 mg/mL, in ACN:MeOH 50:50), 2,4-dinitrotoluene (98.3% purity, 1 mg/mL in ACN:MeOH 50:50), 2,4,6-trinitrotoluene (98.9% purity, 1 mg/mL, in ACN:MeOH 50:50), 4-amino-2,6-dinitrotoluene (98.4% purity, 1 mg/mL, in ACN:MeOH 50:50), and 2-amino-4,6-dinitrotoluene (97.8% purity, 1 mg/mL, in ACN:MeOH 50:50). Isotopically labeled TNT (^13^C_7_, 99%; ^15^N_3_, 98%, 1 mg/mL in benzene, wetted with > 33% H_2_O) was purchased from Cambridge Isotope Laboratories, Inc, Andover, USA and used as internal standard. Acetonitrile (UHPLC-grade, purity ≥ 99.97%), Methanol (LC–MS grade, purity ≥ 99.95%), and Water (LC–MS grade) were purchased from Th. Geyer (Renningen, Germany) and used without further purification. CHROMABOND^®^ Easy polystyrene-divinylbenzene-copolymer reversed-phase solid-phase extraction columns 80 μm, 3 mL/200 mg and 1 mL/30 mg (Macherey Nagel, Düren, Germany) were used. Ultrapure water (18.2 MΩ cm) was prepared on site with a Veolia ELGA Purelab Flex system (Veolia Water Technologies Deutschland GmbH, Celle, Germany). Chemcatcher^®^ 52 mm housings, HLB-L receiving disks and PES membranes were ordered from T.E Laboratories Ltd, Tullow, Ireland.

#### Water sample preparation

Water samples were prepared as described in Bünning et al. (2021). Columns were thawed in the laboratory, dried in a mild vacuum for 0.5 h and eluted with 4 mL ACN. Eluates were concentrated to 1 mL in a RVC 2–25 CDplus rotary vacuum concentrator (Martin Christ Gefriertrocknungsanlagen GmbH, Osterode, Germany) and stored in 1.5-mL amber autosampler vials at − 20 °C until GC–MS/MS measurement. For LC–MS/MS measurements, 0.25 mL of each sample was transferred to a second vial and filled with 0.75 mL LC–MS grade water (containing 0.5-mM ammonium acetate). Samples taken up to August 2020 were analyzed by GC–MS/MS, samples from September 2020 onwards with GC–MS/MS and LC–MS/MS.

#### Blue mussel sample preparation

Mussels were prepared according as previously published (Bünning et al. 2021). Three mussels of every position and depth were thawed, homogenized, freeze dried (Alpha 2–4 LSCplus freeze drier, Martin Christ Gefriertrocknungsanlagen GmbH, Osterode, Germany) and extracted three times with 1 mL ACN. Extracts were filled up to 25 mL with ultrapure Water and given on SPE columns under mild vacuum. Columns were dried and eluted with 4 mL ACN. The eluates were further processed as described under “Water sample preparation” and measured by GC–MS/MS and LC–MS/MS.

#### Passive sampler preparation

The frozen HLB-L-disks and PES membranes were separated, transferred individually to 15 mL polypropylene tubes, and dried overnight in an Alpha 2–4 LSCplus freeze drier (Martin Christ Gefriertrocknungsanlagen GmbH, Osterode, Germany). ACN was added until the membranes were completely covered. The extraction was carried out for 30 min at 4 °C with constant shaking. After removal of the membranes, the extracts were concentrated to 1 mL and processed for measurement by GC–MS/MS and LC–MS/MS as described under “Water sample preparation”.

#### GC–MS/MS measurements

The GC–MS/MS measurements were performed as described in Bünning et al. (2021), using a Thermo Scientific TSQ8000 Evo triple-quadrupole GC–MS/MS system equipped with a Trace1310 gas chromatograph with TraceGold TG-5MS amine columns (15 m × 0.25 mm × 0.25 μm) and a TriPlus 100 LS autosampler. The water and passive sampler samples were analyzed using the splitless method described therein, with tapered quartz wool splitless inection port liners. The mussel samples were measured using a PTV injector with the 5-µL large volume injection method. Measured compounds included 1,3-DNB, 2,4-DNT, TNT, 4-ADNT, and 2-ADNT. The device parameters for both types of injections are listed in Table 3 and the retention times and transitions are given in Table 4.

**Table 3:** GC–MS/MS programs for splitless and large volume injections

	Splitless		Large volume injection
Injector	Split-/splitless		Programmable temp. vaporization
Inlet liner	Glass wool		Glass wool
Injection volume	1 µL		5 µL
Injection temperature	230 °C		70 °C, (0.18 min, 50 mL/min) 5 °C/s to 240 °C (1.5 min, no split) 240 °C (5 min, 200 mL/min)
Column flow	1.5 mL/min		1.2 mL/min
Oven temp	100 °C (0.20 min), 30 °C/min to 220 °C (0.30 min), 80–280 °C (1 min)		100 °C (1 min), 35 °C/min to 220 °C (0.7 min), 70–280 °C (1 min)
Total run time	6.25 min		6.99 min
Transferline temp	250 °C		
Ion source temp	300 °C		
Ionization method	EI

**Table 4:** GC–MS/MS retention times and quantitative (Q) and qualitative (q) SRM transitions of the compounds examined

Compound	Retention time (min)	Precursor ion (*m*/*z*)	Quantitation ion (*m*/*z*)	Collision energy (eV)	Type
1,3-DNB	2.90	122.0	75.0	12	Q
		168.0	75.0	20	q
		168.0	122.0	8	q
2,4-DNT	3.26	165.0	63.1	22	Q
		165.0	90.1	16	q
		165.0	118.1	8	q
TNT	3.96	210.0	164.1	6	Q
		164.0	90.1	10	q
		180.1	76.1	12	q
4-ADNT	4.88	197.0	180.1	6	Q
		180.0	163.1	8	q
		163.0	78.0	14	q
2-ADNT	5.05	197.0	180.1	6	Q
		180.0	133.0	6	q
		180.0	67.0	12	q

#### LC–MS/MS measurements

LC–MS/MS measurements were performed using a Sciex QTrap5500 triple quadrupole mass spectrometer with a Turbo V electrospray ionization source and a UHPLC system consisting of a Shimadzu Nexera LC-40D XS quaternary pump with degasser and an Agilent 1200 G1316A column oven, equipped with an Raptor Biphenyl 1.8-μm column (150 mm × 2.1 mm) and a pre-column (Maser et al. 2024). Measurements started with a 5 min isocratic phase with a ratio of 40% H_2_O (containing 2.5 mM ammonium acetate) and 60% MeOH at 0.25 mL/ min and 35 C, then increased to 95% MeOH by minute 6, and maintained this ratio for 6 min. Retention times and transitions are given in Table 5, and method specific detection and quantification limits in Table 6.

**Table 5:** LC–MS/MS retention times and transitions of the compounds examined

Compound	Retention time [min]	Precursor ion (*m*/*z*)	Quantitation ion (*m*/*z*)	Collision energy (eV)
RDX	2.39	281.0	46.0	− 33
HMX	2.11	355.0	46.0	− 38
TNT	9.04	226.0	46.0	− 50
^13^C^15^N-TNT	9.06	236.0	47.0	− 62
4-ADNT	4.36	196.0	149.0	− 19
2-ADNT	4.59	196.0	46.0	− 56

**Table 6:** Method specific limits of detection of the GC–MS/MS and LC–MS/MS methods

Explosive	GC–MS/MS			LC–MS/MS		
	LoD [fg/µl]	LoQ [fg/µl]	*R*^2^	LoD [fg/µl]	LoQ [fg/µl]	*R*^2^
1,3-DNB	32	105	0.9644	–	–	–
2,4-DNT	10	33	0.9934	–	–	–
TNT	47	155	0.9878	131	430	0.9730
4-ADNT	8	26	0.9959	124	412	0.9764
2-ADNT	11	37	0.9919	83	274	0.9893
RDX	–	–	–	97	320	0.9856
HMX	–	–	–	87	287	0.9884

### Statistical data analysis

All individual samples were measured in duplicate. The evaluations of the GC–MS/MS measurements were performed in Chromeleon 7.2.10, and those of the LC–MS/MS measurements in Multiquant 3.0.3. The EC concentrations of the individual sample vials in ng/mL were calculated based on the integral areas of the peaks using standard curves derived from the results of the co-measured standard dilutions. All substance peaks were checked individually, and, if necessary, manually reintegrated. The data processing and conversion to ng/L for water samples, ng/PS for passive samplers, and ng/g dry weight were performed in Microsoft Excel 2021. The statistical analysis of the measurements and the presentation of the results as box plots were carried out in RStudio 2024.12.0, based on R Version 4.4.2. The comparison of concentrations between the different sampling positions was performed using the non-parametric Kruskal–Wallis test with Dunn’s test as a post-hoc analysis. The Mann–Whitney-Wilcoxon test was used as a non-parametric alternative to the *t* test for comparing the two depths at a position. The significance levels were set at *p* < 0.05, *p* < 0.01 and *p* < 0.005.

## Results

### EC concentrations of the monthly routine water-sampling program

**Fig. 4:**
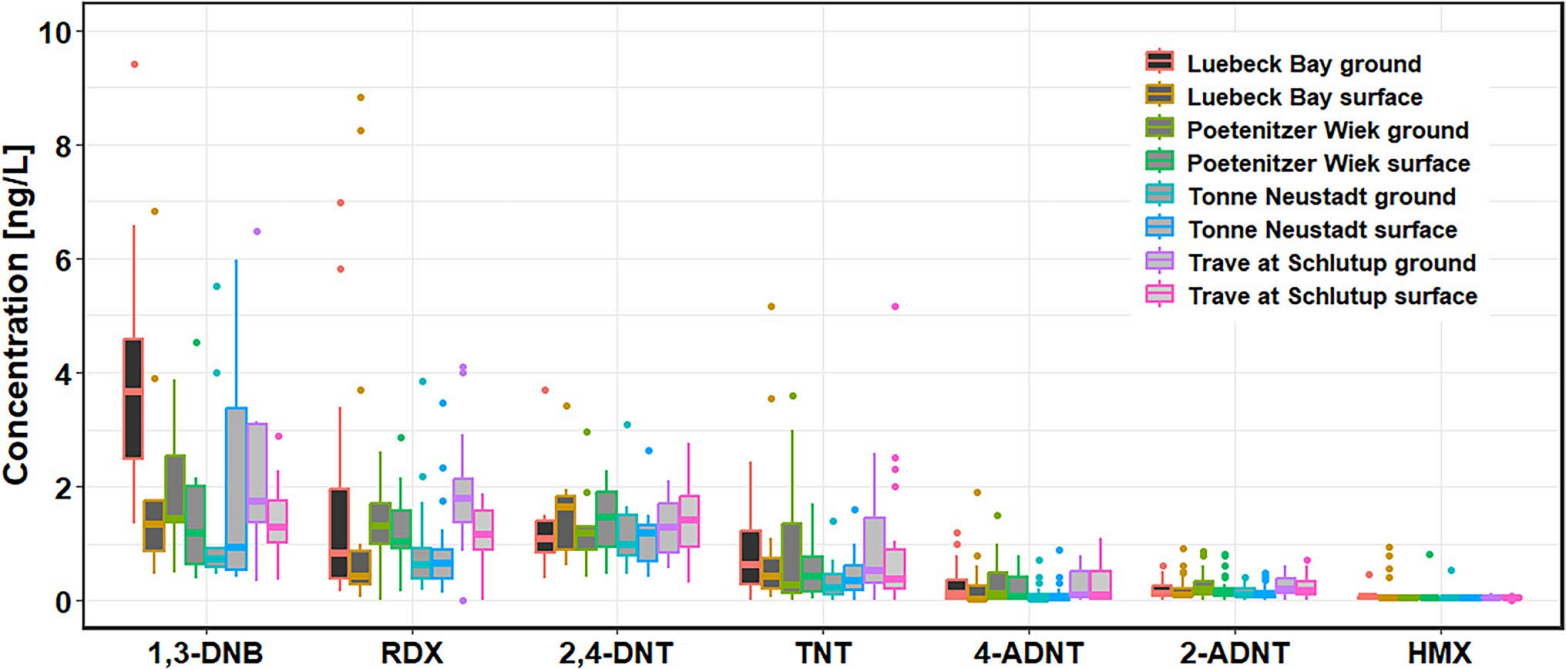
Results of the water samples from the monthly water-sampling program, carried out by the LLUR during their routine monitoring cruises

Energetic compounds were detected in every single water sample from the Bay of Luebeck. However, concentrations greater than 1 ng/L were observed on average only for 1,3-DNB and 2,4-DNT at all sites, and for RDX at all sites except Neustädter Bucht—Tonne Neustadt (Fig. 4). For TNT concentration outliners up to 5.2 ng/L were observed. However, on average, all sample concentrations at all four positions and at both depths were below 1 ng/L. Only sporadically were the concentration differences between the sampling positions significant (TNT: Tonne Neustadt (TN) vs Trave at Schlutrup (TaS): *p* < 0.05; 4-ADNT: TN vs TaS: *p* < 0.05; 2-ADNT: TN vs TaS: *p* < 0.01; RDX: Luebeck Bay (LB) vs Poetenitzer Wiek (PW): *p* < 0.05, LB vs TaS: *p* < 0.005, LB vs TaS: *p* < 0.01, TN vs TaS: *p* < 0.005). The concentration differences between the bottom and the water surface were significant only for RDX at the position Trave at Schlutup with *p* < 0.05.

### Results of blue mussel, passive sampler and water monitoring at specific dump sites

**Fig. 5:**
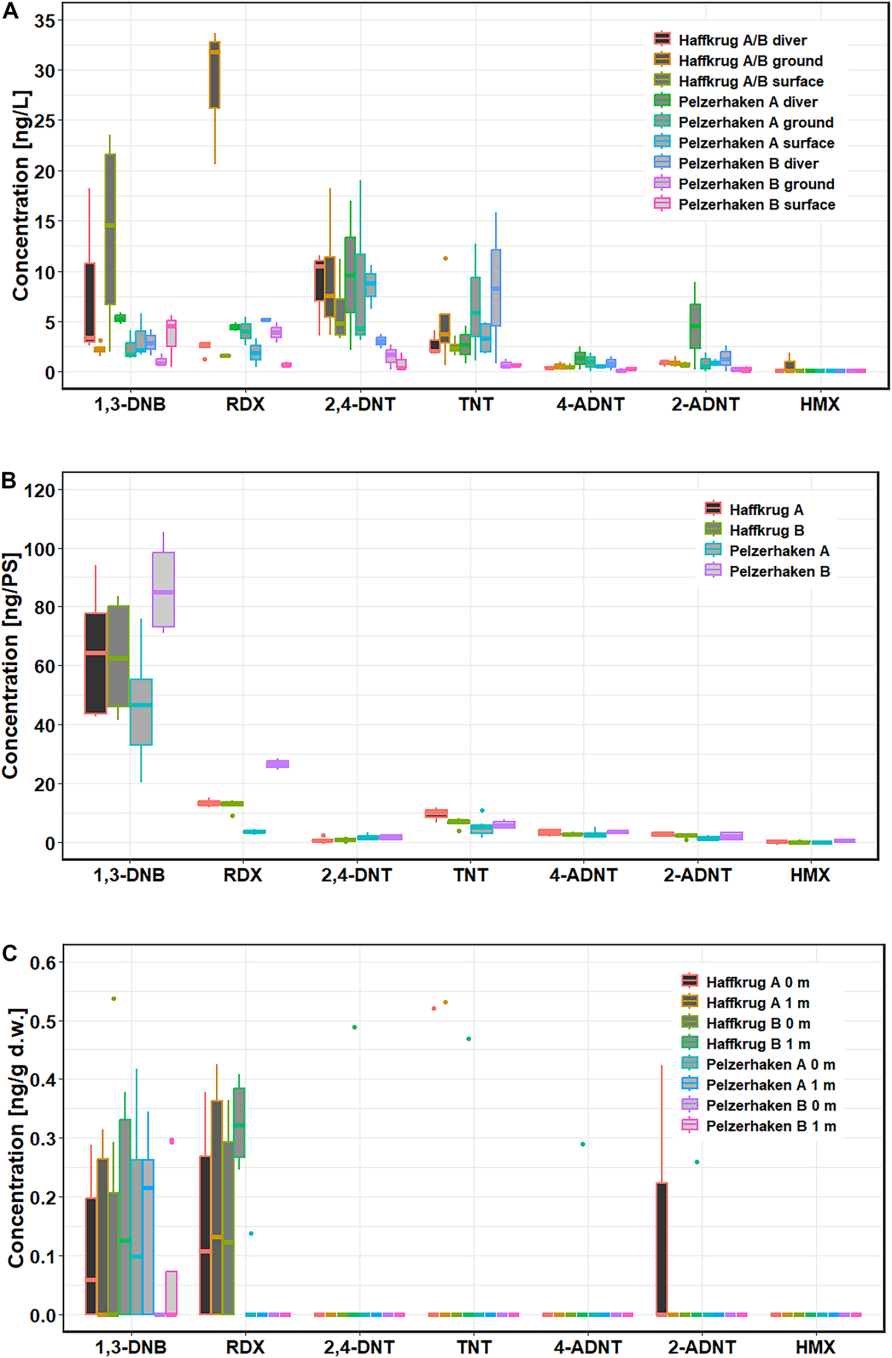
Results of the **A** water, **B** passive sampler and **C** blue mussel samples from the mooring monitoring

In the water samples taken at the mooring positions, EC concentrations were slightly higher compared to the samples from the monthly routine program (Fig. 5A). Overall, 1,3-DNB (max 18.2 ng/L; mean 5.3 ng/L), RDX (max 33.6 ng/L; mean 9.1 ng/L), 2,4-DNT (max 9.5 ng/L; mean 3.3 ng/L) and TNT (max 15.8 ng/L; mean 3.5 ng/L), 4-ADNT (max 2.5 ng/L; mean 0.6 ng/L), 2-ADNT (max 8.9 ng/L; mean 1.1 ng/L), and HMX (max 1.9 ng/L; mean 0.17 ng/L) were detected. The water samples collected by divers directly at the munition objects tended to show slightly higher concentrations than those taken from the ship. However, no statistical significance could be determined.

Compared to the water samples, disproportionately high amounts of 1,3-DNB were measured in the passive samplers with up to 105.4 ng/PS at the position Pelzerhaken B (Fig. 5B). All other EC were below 20 ng/PS, with the exception of RDX at Pelzerhaken B, with a maximum of 28.7 ng/PS. TNT concentrations ranged from 5.1 ± 2.7 ng/PS in Pelzerhaken B to 9.6 ± 1.9 ng/PS in Haffkrug. Statistically significant differences in passive sampler concentrations were only found for TNT between Haffkrug A vs Pelzerhaken A (*p* < 0.05), and for 1,3-DNB, RDX, and HMX between Pelzerhaken A and Pelzerhaken B (1,3-DNB: *p* < 0.05, RDX: *p* < 0.05, HMX: *p* < 0.05).

EC were only detected in individual mussels (Fig. 5C). While 1,3-DNB was detected at all four locations with a maximum quantity of 0.54 ng/g dry weight in Haffkrug B at the bottom, RDX was detected only in samples from Haffkrug, with the exception of a single mussel sample from Pelzerhaken A. TNT, as well as its metabolites 2- and 4-ADNT, were also found only in a few individual samples from Haffkrug (Fig. 5C), with no differences between mussels on seafloor and those from 1 m above seafloor. The production byproduct 2,4-DNT was only detected in a single sample from Haffkrug, while HMX was not found in any of the examined mussels. No statistically significant differences were found for any of the measured EC between the various positions or the two heights above ground in the mussels.

## Discussion

This pilot study of the Bay of Luebeck demonstrates that routine water sampling and blue mussel monitoring at specific dumping sites are principally suitable for the surveillance of energetic compounds in munition contaminated waters. However, an issue was the low oxygen levels in the Bay of Luebeck, particularly during the summer months (Lutterbeck 2019). When recovering the mussels deployed at the Pelzerhaken A position in August 2020, only empty shells were retrieved, indicating that the mussels had died. This disadvantage with living species can principally be compensated for by using passive sampling systems. Apart from natural obstacles such as weather, water currents and poor visibility underwater, mooring systems could be subject to being dragged by fishing nets. This could be the reason, why, in our case, two moorings could not be recovered on separate occasions: An original mooring deployed in the Haffkrug-Area in August 2020 and a further one in Pelzerhaken B in March 2021. An additional limiting factor in our special case was that some of the originally planned cruises in the years 2020 and 2021 had to be canceled due to restrictions from the Covid-19 pandemic.

EC were detected in all water samples of the monthly sampling campaign. This is consistent with previous publications from the German Baltic Sea, where EC were found at every investigated location (Beck et al. 2025; UDEMM et al. 2019). No seasonal trend regarding the concentration of EC could be identified in the monthly water samples. Similarly, there was no general trend between the samples taken at the seafloor and those collected 1 m below the water surface, which indicates strong vertical mixing within the water column. Only for 1,3-DNB and RDX measured values near the seafloor appear to be slightly elevated on average, but was only determined to be statistically significant for RDX at the sampling location Trave at Schlutup.

Particularly striking in the Bay of Luebeck is the increased occurrence of 1,3-DNB in relation to TNT. Only in four out of around one hundred water samples was the 1,3-DNB concentration lower than the TNT concentration. In all other samples, 1,3-DNB was measured at levels 2–34 times higher than TNT (1,3-DNB: 0.3–9.4 ng/L; TNT: < LOD-5.2 ng/L). In the passive samplers, 1,3-DNB was measured at levels 3.9–15.8 times higher than TNT (1,3-DNB: 20.4–105.5 ng/PS; TNT: 1.5–11.8 ng/PS). The nitroaromatic 1,3-DNB is an intermediate product in the production of the explosives TNT and trinitrobenzene (TNB) and was used as a readily available substitute for TNT in varying percentages in the filling of bombs during World War II. While 1,3-DNB almost always appears together with TNT in the environment, it is usually present in much lower concentrations than TNT. For example, in the munition dumping area of the Oosterschelde estuary—a North Sea region in the Netherlands, the ratio of 1,3-DNB to TNT in the analyzed water ranged from 3.9 to 26.8% (1,3-DNB: 0.2–7.8 ng/L; TNT: < LOD–56.7 ng/L) (Den Otter et al. 2023). In the area with hexanite (German: “Schiesswolle” of the Kolberger Heide dumping ground with uncovered explosive chunks, the proportions were even lower, ranging between 0–7.9% (1,3-DNB: 0–2.0 ng/L; TNT: 6.8–1346.1 ng/L) in water and 0.18% and 5.7% in passive samplers (1,3-DNB: 101.1–1499.0 ng/PS; TNT: 2183.5–119,754.4 ng/PS). Other studies reported 1,3-DNB to TNT ratios of 5.9/14.2 µg/L (Halifax Harbor, Canada (Rodacy et al. 2001)), 0.008/7.5 µg/L (Bahia Salina del Sur, Puerto Rico (Rosen et al. 2018)), 23.4/105 µg/L (Isla de Vieques Bombing Range, Puerto Rico (Porter et al. 2011)), 0.18/0.17 µg/L (Lake Mjøsa, Norway (Rossland et al. 2011)), and 10/50 µg/L (Kolberger Heide, Germany (Beck et al. 2019)).This atypical ratio in favor of 1,3-DNB compared to TNT has already been observed in previous investigations along the German Baltic Sea coast, with 30-fold higher 1,3-DNB concentrations in the water of the Bay of Luebeck compared to the Kolberger Heide (UDEMM et al. 2019). The reason for this, however, remains unclear. It is possible that multiple exposed explosive objects, presumably warheads from the Fieseler Fi 103 flying bomb, are the source. A significant dissolution of their uncovered, sponge-like explosive material was observed between March 2023 and 2024 (Greinert et al. 2024).

As could be expected, higher EC concentrations were measured in the water at the four moorings compared to the routine monthly water samples, albeit these differences were only marginal. Overall, EC water concentrations in the Bay of Luebeck from our study correspond to findings from other munition dumping sites with concentrations in the single- or double-digit ng/L, and with higher concentrations reported in close proximity to solid munitions material: Eastern Scheldt, Netherlands (Den Otter et al. 2023), Sejerø Bay, Denmark (Maser et al. 2023a), Kolberger Heide, Germany (Beck et al. 2019; Esposito et al. 2023; Gledhill et al. 2019), and at a shipwreck in the North Sea of Belgium (Maser et al. 2023b).

Blue mussels in our study showed very low levels of contamination. Only 1,3-DNB and RDX were regularly detected in very low concentrations, while TNT and its metabolites 2- and 4-ADNT were found only in individual mussels. In previous studies, much higher tissue concentrations were found at other dumping sites close to munition objects. In the Kolberger Heide, up to 10 ng/g wet weight (approximately 100 ng/g dry weight) of the TNT metabolite 4-ADNT were detected in the tissue of mussels, deployed directly at corroded moored mines (Appel et al. 2018), and up to 150 ng/g wet weight (~ 1500 ng/g dry weight) of 4-ADNT were measured near free-lying chunks of hexanite (German: “Schiesswolle”) (Strehse et al. 2017). In the area around the ship wreck of the V1302 (“John Mahn”), which sank in the North Sea during World War II,TNT concentrations in mussels reached up to 3 ng/g dry weight (Maser et al. 2023b). Interestingly, concentrations of 1,3-DNB, 4-ADNT, and 2-ADNT above the detection limit were found onwards from the year 2017 until today in wild blue mussels collected since 1992 by the Environmental Specimen Bank from the Darssßer Ort area, located further east of the Bay of Luebeck. However, these concentrations were below the quantification limit (Strehse et al. 2023). Similar findings were reported in the same study for two other locations at the coastline of the North Sea region—Eckwarderhoerne located in the Lower Saxony Wadden Sea and the island of Sylt (Koenigshafen) located in the Schleswig–Holstein Wadden Sea (Strehse et al. 2023).

The question now is what the results of our study mean for the marine environmental ecology and the safety of human seafood consumers. Literature reviews of the effects of EC to aquatic organisms were first provided by Talmage et al. (1999), and later by Juhasz and Naidu (2007), Nipper and Lotufo (2009) and Lotufo (2013). Lotufo et al. (2013, 2017, 2021), Voie and Mariussen (2017), Beck et al. (2018), and Barbosa et al. (2023) provided comprehensive summaries and concise overviews on the toxicity of EC to aquatic biota. As a conclusion from all of these studies it appears that EC toxicity benchmarks values for aquatic biota are in the μg/L to mg/L ranges when considering acute toxicity studies that were performed under short-term laboratory conditions.

However, the situation with submerged munitions is much more complex in the oceans and involves the following important aspects. Firstly, a distinction must be made between acute (lethal) and sublethal toxicity. Acute toxicity is considered a somewhat different issue with mortality as an endpoint, while sublethal toxicity has an impact on the general health of marine organisms. A deteriorating state of health not only reduces the longevity of individuals, but can also negatively influence the population dynamics of marine organisms. Together with other anthropogenic influences (microplastics, pharmaceuticals, endocrine disruptors, pesticides), EC from world war relics can significantly affect sea food stocks. For example, Schuster et al. (2021) observed significant responses of the antioxidant defense system in blue mussels (*Mytilus* spp.) at the biochemical and cellular biomarker level, while Koske et al. (2019) found lethal and sublethal effects of TNT and its transformation products 2-ADNT and 4-ADNT to zebrafish embryos (*Danio rerio*) in a 120-h exposure scenario. Lethal concentrations (LC_50_) were 4.5 mg/l for TNT, 13.4 mg/l for 2-ADNT, and 14.4 mg/l for 4-ADNT, while sublethal effects such chorda deformation and genotoxicity were induced by all three compounds already at 0.1 mg/l as lowest tested concentration. In field studies, low levels of EC in the 1–10 ng/L range have been occasionally detected directly adjacent to submerged munitions (Gledhill et al. 2019; Maser et al. 2023a), whereas higher TNT concentrations of up to the two-digit µg/L range have been observed near exposed explosive solids (Beck et al. 2019), thereby reaching concentration that might impact the marine life.

A second aspect is the time of exposure to contaminants. While laboratory studies are generally performed with relatively high EC concentrations and short exposure periods, the biota in the marine dumping sites are usually exposed to lower concentrations but lifelong. In the southwestern Baltic Sea, Beck et al. (2025) detected at least one EC compound in nearly every water sample in concentrations ranging from sub-pmol/L up to several thousand pmol/L. In this area, Blue mussels (*Mytilus* spp.) had been deployed as a biomonitoring system for the presence of EC near corroding mines (Strehse et al. 2017, 2020; Appel et al. 2018; Maser and Strehse 2020). After a deployment period of 93 days, mussels bioaccumulated TNT and its transformation products and a decrease in shell growth in combination with weight loss were observed. Moreover, a statistically significant induction of the carbonyl reductase gene in the tissues of *Mytilus* spp. placed in cages adjacent to a chunk of explosive material was shown after a 58-day exposure (Strehse et al. 2020). The enzyme carbonyl reductase plays an important role in the detoxification of TNT-caused carbonyl stress, as has been proven in lab studies with *Mytilus* spp. (Strehse et al. 2020) and *Daphnia magna* (Jacobsen et al. 2022, reviewed in Adomako-Bonsu et al. 2024). Whether such chronic toxic effects can be detected in the biota living in the Bay of Luebeck remains a question for future studies.

For humans, as consumers of potentially contaminated seafood, such as mussels, health impacts cannot be entirely ruled out. On the one hand, it is important to carefully infer the actual concentrations of energetic compounds present in seafood. On the other hand, the average and actual consumption behavior of different population groups with regard to this EC contaminated seafood is crucial. Calculations based on the mussels collected near the corroded anchor buoys in Kolberger Heide, where an average of 5 ng/g fresh weight (equivalent to about 50 ng/g dry weight) of TNT metabolites were detected (Appel et al. 2018), indicate that a health risk would only arise from a daily and lifetime consumption of several kilograms of these mussels. Only mussels from areas with exposed explosives, containing up to 350 ng/g fresh weight (equivalent to 3500 ng/g dry weight), would pose a health risk, based on the assumption that 39 g of mussels were consumed daily over a lifetime (Maser and Strehse 2021). This latter calculation is based on the average daily intake of fish and seafood of 39 g per person in Germany (FIZ 2017). Since the lifelong daily consumption of 39 g of mussels from the immediate vicinity of chunks of explosives lying without covers on the sea bed is very unlikely, the consumption of mussels from the Baltic Sea can be generally considered as being safe from today’s point of view (Maser and Strehse 2021). Therefore, based on the concentrations measured in the mussels of the Bay of Luebeck (< 1 ng/g dry weight), there is currently no health risk for humans even with daily and lifelong consumption of these mussels (Beck et al. 2022; Maser and Strehse 2021, 2020).

However, the aspect of ongoing corrosion of the metal casings of the munition bodies comes into play. After 80 years of resting on the seabed, there are clear signs that corrosion is progressing continuously and that increasing amounts of EC are entering the marine environment. Hence, as time goes on, not only the marine biota is becoming increasingly endangered, but also the human seafood consumer. As already mentioned above, this temporal aspect was recently shown in a study on mussels from the environmental specimen bank. Here, mussels from years 1985 to 2021 were examined for their EC content. While mussels from the Darßer Ort from years 1992 to 2016 showed no evidence of EC, these were clearly detected in mussels from years 2017 to 2021, and with increasing concentrations (Strehse et al. 2023).

## Conclusion

Over the course of the 4 years, it became apparent that energetic compounds are ubiquitous detectable in the Bay of Luebeck, but, as of today, their concentrations are low. This applies to the entire Bay of Luebeck as well as to the munition dumping areas investigated in the current study and is comparable to other munitions dumping areas like Kolberger Heide in the Baltic Sea or Eastern Scheldt in the North Sea. The marginal difference in EC water concentrations directly at munition dumping sites compared to water samples farer away (from the routine monitoring program), as well as the lack of significant differences in EC concentrations between the water samples taken at the seafloor or below the surface indicates a strong mixing of the horizontal and vertical water body in the Bay of Luebeck. Further, low EC concentrations have been found in blue mussels exposed directly at munition dump sites. Thus, EC concentrations in water and mussels do currently not indicate any risk to the marine aquatic biota, to touristic bathers nor seafood consumers in the Bay of Luebeck area.

However, enhanced release of EC from underwater munitions in the southwest Baltic Sea is certain as corrosion advances. To prevent an increasing contamination of the seas worldwide the remediation of munitions from the sea should begin immediately. Our findings are a promising base for the upcoming clearance operation of the Bay of Luebeck as part of the German government’s immediate action program to remove submerged munitions from the sea. In contrast to blow-in-place disposal (e.g., Maser et al. 2023a), this method of remediation is likely to have low negative environmental impact. To detect an increase in EC pollution during the clearance operation of the munition bodies early enough, extensive monitoring of these activities is required—not only with respect to water samples, which only provide a snapshot of the situation at one location, but also using mussels and passive sampling systems which can record EC-concentrations over longer periods of time.

## Data Availability

Data are publicly available.
